# Comparative Transcriptome Profiles of Human HaCaT Cells in Response to *Gynostemma pentaphyllum* Extracts Obtained Using Three Independent Methods by RNA Sequencing

**DOI:** 10.3390/life13020423

**Published:** 2023-02-02

**Authors:** Won Kyong Cho, Seung Hye Paek, Soo-Yun Kim, Sung Joo Jang, Sak Lee, Hoseong Choi, Yeonhwa Jo, Jeong Hun Lee, Sang Hyun Moh

**Affiliations:** 1 College of Biotechnology and Bioengineering, Sungkyunkwan University, Suwon 16419, Republic of Korea; 2 Plant Cell Research Institute of BIO-FD&C Co., Ltd., Incheon 21990, Republic of Korea; 3 Plant Health Center, Seoul National University, Seoul 08826, Republic of Korea

**Keywords:** *Gynostemma pentaphyllum*, extract, HaCaT, transcriptome, RNA sequencing

## Abstract

*Gynostemma pentaphyllum* (GP) is widely used in herbal medicine. In this study, we developed a method for the large-scale production of GP cells using plant tissue culture techniques combined with bioreactors. Six metabolites (uridine, adenosine, guanosine, tyrosine, phenylalanine, and tryptophan) were identified in GP extracts. Transcriptome analyses of HaCaT cells treated with GP extracts using three independent methods were conducted. Most differentially expressed genes (DEGs) from the GP-all condition (combination of three GP extracts) showed similar gene expression on treatment with the three individual GP extracts. The most significantly upregulated gene was *LTBP1*. Additionally, 125 and 51 genes were upregulated and downregulated, respectively, in response to the GP extracts. The upregulated genes were associated with the response to growth factors and heart development. Some of these genes encode components of elastic fibers and the extracellular matrix and are associated with many cancers. Genes related to folate biosynthesis and vitamin D metabolism were also upregulated. In contrast, many downregulated genes were associated with cell adhesion. Moreover, many DEGs were targeted to the synaptic and neuronal projections. Our study has revealed the functional mechanisms of GP extracts’ anti-aging and photoprotective effects on the skin using RNA sequencing.

## 1. Introduction

Plants have been regarded as a useful source of natural medicines for curing diverse human diseases since ancient times [1]. Plants are rich in phytochemicals that are useful as natural medicines and provide health benefits. Nowadays, natural medicines are more popular because of their beneficial effects and lower prices, compared to high-cost synthetic drug agents, and are widely used for the prevention and treatment of many diseases [2]. Hundreds of plant species have been widely used as natural herbal materials in many industries, such as pharmaceuticals, cosmetics, and nutraceuticals [3].

*Gynostemma pentaphyllum* (Thunb.) Makino, known as Jiaogulan in China, is a dicotyledonous and perennial plant belonging to the Cucurbitaceae family, which includes cucumber, pumpkin, and melon [4]. *G. pentaphyllum* (GP) has a dioecious reproductive system, with distinct individual organisms that produce male or female gametes. GP is an herbaceous vine plant distributed in the United States and Asian countries, such as China, Korea, Japan, Malaysia, and India, and has been traditionally consumed as a food and tea in many countries [5].

Numerous studies have indicated that GP extracts have many beneficial effects, such as antioxidant [6,7], anticancer [8], cholesterol reduction [9], hypoglycemic [10,11], and immunopotentiation effects [12]. GP extract contains a diverse group of substances. Of these, saponins, also known as gypenosides, are the main constituents of GP extracts [13]. Gypenosides from GP extracts are similar to those from ginseng plant species [14,15], and both GP and ginseng extracts have anti-diabetic effects.

Secondary metabolites are the phytochemical components of various pharmaceutical plants [16]. The production of secondary metabolites in plants is often regulated by environmental conditions, such as biotic and abiotic stresses, to maintain plant physiology.

Recently, the development of plant tissue culture techniques and large-scale plant cell culture using bioreactors has facilitated the massive production of plant components, such as secondary metabolites from diverse plants [17]. Plant cell culture techniques are independent of climate and geographical region, and a small amount of initial plant material can lead to large-scale production within a short amount of time using a bioreactor [18]. Furthermore, the quality of the plant material in the bioreactors is uniform, enabling easier quality management.

The composition of plant extracts can vary according to the extraction method [19]. The amount of active ingredients present in plant materials is usually very low. Thus, effective and appropriate methods should be selected to extract the target bioactive natural compounds.

With the rapid development of high-throughput sequencing, RNA sequencing has become a powerful tool for the gene expression analysis of transcriptomes. Moreover, several previous studies have demonstrated that RNA sequencing-based transcriptome approaches are efficient in identifying differentially expressed genes (DEGs) associated with several plant extracts, such as *Leontopodium alpinum* (Edelweiss) [20], *Camellia japonica* [21], *Isatis tinctoria* L. [22], *Ricinus communis* L. [23], and metabolites such as porphyra-334 [24], kaempferol [25], Angelica polysaccharide [26], and plant-derived exosomes [27].

In this study, we established a method for the large-scale production of GP cells using plant tissue culture techniques combined with bioreactors. Furthermore, we conducted transcriptome analyses of HaCaT cells in response to three different GP extracts to identify the key genes related to the effects of GP extracts.

## 2. Materials and Methods

### 2.1. Generation of GP Cells and Suspension Cell Cultivation Using Bioreactors

The GP plant was collected from Sadong-ri, Ulleung-eup, Ulleung-gun, Gyeongsangbuk-do in Korea on July 1, 2020 by Dr. Sang Hyun Moh. The *rbcL* amplicon sequence from the collected GP plant shared 100% sequence identity to that of *Gynostemma pentaphyllum* (Thunb.) Makino (Registration number: NIBRGR0000654599) deposited in the National Institute of Biological Resources (https://species.nibr.go.kr (accessed on 3 July 2022)).

The leaves were soaked in 70% ethanol for 30 s, followed by washing with distilled water. The leaves were shaken in 0.3% sodium hypochlorite (Waco, Osaka, Japan) for 20 min and washed again with distilled water. The leaves were cut into small pieces (0.5 to 1 cm) under aseptic conditions. The early stages of the plant cells were cultured in Murashige and Skoog (MS) medium (M0222, Duchefa, Haarlem, The Netherlands) [28] with plant growth regulators, auxin and cytokinin, in the dark at 25 ± 2 °C. The first induced plant cell was propagated in the same petri dish for 2–3 weeks. From the eighth week, the optimal combination ratio of dichlorophenoxyacetic acid (2,4-D; D0911, Duchefa) was chosen based on the color, shape, and degree of differentiation of the plant cells. The selected plant cell line or callus was propagated in the optimal medium containing 4.4 g/L of MS, 30 g/L sucrose (S0809, Duchefa), 1 mg/L 2,4-D, 2.3 g/L gelrite (G1101, Duchefa) at pH 5.8 in a petri dish. Thereafter, the callus was cultured in a bioreactor at the Plant Cell Research Institute of BIO-FD&C Co., Ltd., Incheon, Republic of Korea.

Freshly prepared plant cell cultures of GP were filtered to harvest the plant cells from the culture media. Thereafter, 100 g each of the cells were transferred to three bioreactors (total volume three liters), with each bioreactor containing 2 L of fresh MS (4.4 g/L, pH 5.8) basal medium supplemented with 30 g/L of sucrose and 1 mg/L 2,4-D.

### 2.2. Treatment of GP Cells with Radiofrequency and Extraction of GP Cells

Three different GP extracts were prepared in this study. The GP extract from the plants, referred to as GP-P, was prepared as follows. GP plants were dried at 60 °C for two days using an agricultural dryer (Kiturami, KED-S12D1, Seoul, Republic of Korea). Subsequently, the dried GP plants were heat extracted with distilled water at 121 °C for 15 min. The extracts were stored at 4 °C. To prepare GP calli (GP-C), the plant cells (callus) from the tissue culture were further cultured for six days. To prepare the GP callus treated with radiofrequency (GP-CR), the plant cells were subjected to radiofrequency at 360 kHz for 20 min per day, and the treatment was repeated for three days. Thereafter, the cells treated with or without radiofrequency were harvested using a non-woven fabric filter. The harvested plant cells were subjected to heat extraction using distilled water at 121 °C for 15 min. For all three extract preparations, 2 g/L of the sample was used for extraction. Solids were removed by filtration through a mesh after heat extraction.

### 2.3. Analyses by High-Performance Liquid Chromatography (HPLC) and Tandem Mass Spectrometry (MS/MS)

Chromatography experiments were performed using an HPLC system (1260 Infinity II, Agilent, Santa Clara, CA, USA). Water and acetonitrile (HPLC grade) were purchased from Samchun Pure Chemicals (Pyeongtaek, Republic of Korea), and trifluoroacetic acid of Alfa Aesar (Schiltigheim, France) was used. For the identification of each individual peak appearing in the HPLC chromatogram, authentic compounds (assay ≥ 98%) of adenosine (#A9251), guanosine (#G6752), phenylalanine (#P2126), tryptophan (# T0254), tyrosine (#T3754), and uridine (#U3750) were purchased from Sigma-Aldrich (St. Louis, MO, USA). The analytical column was a Shim-pack GIS C18 (5 μm, 4.6 × 250 mm, Shimadzu, Kyoto, Japan), and the mobile phase was a mixture of eluent A (0.1% trifluoroacetic acid in water) and B (0.1% trifluoroacetic acid in acetonitrile). Elution was performed with a linear gradient of eluent A and B under the following conditions: 0→5 min, 0→0% B; 5→75 min, 0→75% B; 75→77 min, 75→95% B; 77→82 min, 95→95% B; 82→84 min, 95→0% B and 84→95 min 0→0% B. The flow rate was 1.0 mL/min, the injection volume was 20 μL, and detection was performed at 210 nm. All samples were filtered with a 0.45 μm syringe filter (PTFE, Advantec, Tokyo, Japan) before injection. To identify peaks from the HPLC results, we conducted MS/MS using the 3200 QTRAP system (AB Sciex, Framingham, MA, USA), as described previously [29].

### 2.4. Cultivation and Differentiation of the Human Cells

Human HaCaT keratinocytes (Cat. No. CRL-2404), obtained from the American Type Culture Collection (ATCC) (Manassas, VA, USA), were cultured in Dulbecco’s Modified Eagle’s Medium (DMEM; Welgene, Gyeongsan-si, Republic of Korea) supplemented with 10% heat-inactivated fetal bovine serum (FBS) (Thermo Fisher Scientific, Waltham, MA, USA) and 100 U/mL penicillin/streptomycin mixture (Thermo Fisher Scientific) at 37 °C in a humidified atmosphere containing 5% CO_2_. The cells were passaged at 80–90% confluence. mycoplasma contamination is very common in cell cultures. To confirm the presence of mycoplasma in the cell cultures, we performed PCR using specific primers (MGSO and GPO-3) for the *Mycoplasma* species amplifying a PCR product with 270 bp in length as described in the previous study [30]. As a result, we confirmed that our HaCaT cell cultures were free from the mycoplasma contamination. We worked with HaCaT cells carefully under aseptic conditions to prevent contamination. For the treatment of HaCaT cells, 0.1% of the GP extract from either of the three different extraction conditions (GP-P, GP-C, and GP-CR) was added to the cell culture medium and incubated for 24 h; the control samples were treated with an equal volume of distilled water.

### 2.5. Total RNA Isolation, Library Preparation, and RNA Sequencing

For the transcriptome analyses of HaCaT cells in response to the treatments (GP-P, GP-C, and GP-CR), the cells were seeded at a density of 1 × 10^6^ cells per well in a six-well plate and incubated for 24 h. They were then treated with 0.1% of either of the three extracts (GP-P, GP-C, and GP-CR) or distilled water (control) for 24 h. Three biological replicates were harvested for each condition. Total RNA was isolated from the cells using TRIzol reagent (Invitrogen, Waltham, MA, USA) according to the manufacturer’s protocol, and the quality of the extracted total RNA was measured using Bioanalyzer 2100 (Agilent, Santa Clara, CA, USA). RNA samples were quantified using a Thermo Scientific Multiskan GO microplate spectrophotometer (Fisher Scientific Ltd., Vantaa, Finland), and purity was assessed using the ratio of absorbances at 260 nm and 280 nm (A ratio between 1.8 and 2.0 was considered acceptable). Total RNA with an RNA integrity number (RIN) value ≥ 7 was used for library preparation. RNA sequencing libraries were prepared using the TruSeq Stranded mRNA LT Sample Prep Kit (Illumina, San Diego, CA, USA) according to the manufacturer’s instructions. Twelve libraries with their respective indices were paired-end sequenced using the HiSeq X system (Illumina).

### 2.6. Mapping and Identification of DEGs

The raw data were deposited in the NCBI SRA database under the PRJNA903215 accession number. Raw sequence reads were mapped to the human reference genome (GRCh38) (https://www.ncbi.nlm.nih.gov/genome/guide/human/ (accessed on 10 October 2022)) using BBMap ver. 39.00 with the default parameters. HTSeq ver. 2.0.1 was used to quantify the gene expression for each gene using the GRCh38 general transfer format (GTF) (Homo_sapiens.GRCh38.108.chr.gtf) derived from Ensemble [31]. The number of mapped reads in each transcript was used to identify DEGs using DESeq2 ver. 1.38.3 in DEBrowser v1.24.1 [32]. We compared the GP extract-treated HaCaT cells with those treated with distilled water (control). The number of reads was normalized using the MRE normalization method without any correction. By applying a fold change (FC) of more than four times and *p*-values less than 0.01, we identified DEGs for each comparison. For the GP-all comparison, all nine datasets from the GP extract-treated samples were compared with the three control datasets.

### 2.7. Gene Set Enrichment Analyses

The DEGs identified from GP-all were divided into upregulated and downregulated genes. The gene symbols in each group were used for gene set enrichment analyses using the WebGestalt program [33]. WebGestalt uses an overrepresentation analysis (ORA) against the Gene Ontology (GO) functional database (Release on 1 January 2019), which is further divided into three categories: biological processes, cellular components, and molecular functions. First, we determined the number of genes mapped to GO Slim. Genes annotated to the selected functional categories were used for the enrichment analysis of the human genome. The minimum and maximum number of IDs in the category were set to 5 and 2000, respectively. Bonferroni and the top 100 were used for the FDR method and significance level, respectively. Further, only the identified functional gene sets with a *p* < 0.05 were selected.

## 3. Results

### 3.1. Production of Plant Cells Using Plant Tissue Culture Technology

**Figure 1:**
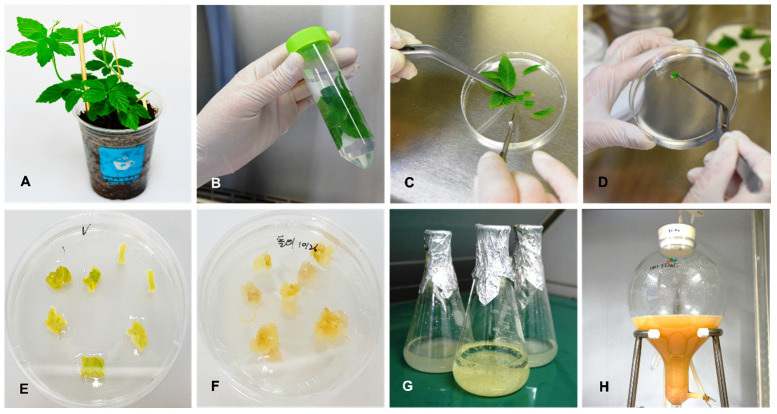
Generation and cultivation of GP cells from GP leaves using tissue culture and bioreactors. (**A**) GP plant used for tissue culture. (**B**) Sterilization of the GP leaves. (**C**) Cutting of the GP leaves. (**D**) Transfer of the GP leaves on the plate containing MS medium. (**E**) Induction of the GP callus. (**F**) Propagation of the GP callus. (**G**) Suspension cell cultivation of the GP callus in liquid medium. (**H**) Mass cultivation of the GP cells in a bioreactor. GP, *Gynostemma pentaphyllum*; MS, Murashige and Skoog.

The prepared GP leaves were sterilized and used for the callus induction (Figure 1). The induced GP callus was used for suspension cell cultivation in a liquid medium. Finally, GP cells were mass-cultivated using bioreactors.

Three different GP extracts, such as the GP extract from the plants (GP-P), the extract from the GP plant cells (callus) (GP-C), and the GP plant cells treated with radiofrequency (GP-CR) were prepared in this study. Next, we conducted an HPLC analysis of three different extracts (Figure S1). The chromatograms showed the representative six highest peaks in all three GP extracts (Figure S1A). Interestingly, the six peaks were much higher in GP-C and GP-CR than in GP-P. We examined the six peaks in each GP extract using MS/MS analysis (Figure S1B)and identified uridine (peak 1), adenosine (peak 2), guanosine (peak 3), tyrosine (peak 4), phenylalanine (peak 5), and tryptophan (peak 6) by comparison to the MS/MS of the authentic compounds (Figure S1C).

Total RNA samples extracted from HaCaT cells treated with either extract or distilled water (control) were used to prepare 12 libraries for RNA sequencing (Table 1). The read numbers ranged from 49,818,356 (GP-C3) to 32,703,588 reads (GP-CR3) (Table 2). The GC content of the raw data was higher than 50% in all 12 libraries. GP-P1 had the highest GC content (51.19%), and GP-CR1 had the lowest (50.48%). The quality scores of 20 (Q20) and 30 (Q30) in all libraries were higher than 98% and 94%, respectively, indicating that the sequenced raw data was high quality.

### 3.2. Mapping and Identification of DEGs

**Figure 2:**
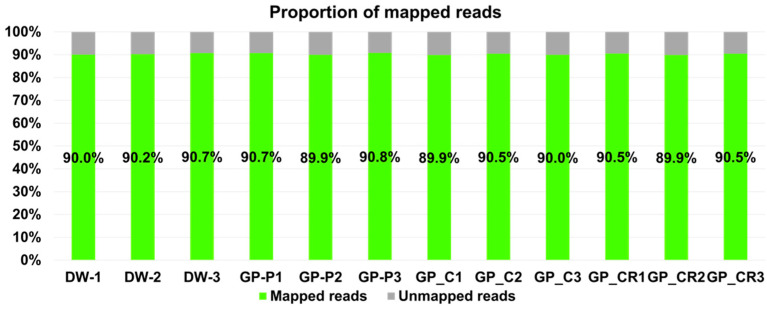
Proportion of mapped reads on the human reference genome in each library.

Raw data were mapped onto the human reference genome (GRCh38) using the BBMap program with default parameters. The proportion of mapped reads in each library ranged from 90.7% to 89.9% (Figure 2). Approximately 10% of the sequenced reads in each library were not associated with human transcripts.

**Figure 3:**
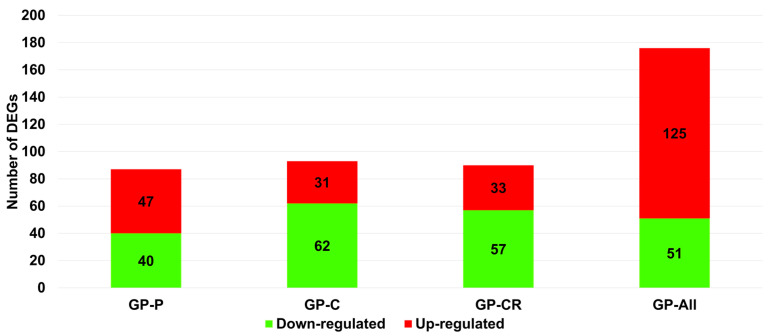
Number of DEGs identified in each condition. Green and red colors indicate down- and upregulated genes, respectively. Four comparisons were conducted: GP-P (GP extract from the plant leaves), GP-C (GP extract from the callus), GP-CR (GP extract from the callus treated with radiofrequency), and GP-all (Combination of all three GP extracts). GP, *Gynostemma pentaphyllum*; DEGs, differentially-expressed genes.

We identified DEGs by comparing the GP extract-treated and control samples. The numbers of DEGs identified under treatments with the three extracts were very similar (Figure 3). Among the extracts, GP-C (93 DEGs) showed the highest number of DEGs, followed by GP-CR (90 DEGs) and GP-P (87 DEGs). In addition, we combined all GP extract-treated samples, referred to as GP-all, and compared them to the control conditions, resulting in the identification of 176 DEGs. In both GP-P and GP-all conditions, the number of upregulated genes was higher than that of downregulated genes. The number of upregulated genes (125 genes) was almost three times that of the downregulated (51 genes) in GP-all. In contrast, the number of downregulated genes was much higher than that of the upregulated genes in the GP-C and GP-CR conditions.

### 3.3. DEGs in Response to GP Extracts

**Figure 4:**
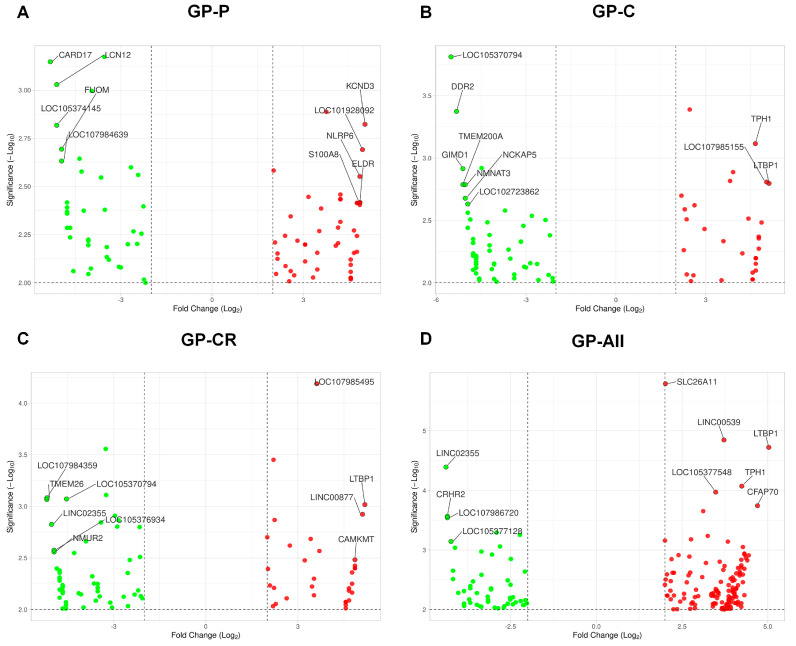
Volcano plot displaying the top ten differentially-expressed genes in each condition. Differentially-expressed genes were identified in four different groups such as GP-P (**A**), GP-C (**B**), GP-CR (**C**), and GP-All (**D**) as compared to the control group. Green and red colors indicate down- and upregulated genes, respectively. The top ten ranking genes are indicated by respective gene names based on the Manhattan distance using the VolcaNoseR program.

We selected the top ten significant DEGs in each condition based on the Manhattan distance (Figure 4). Among the 87 DEGs in GP-P (Table S1), the top ten DEGs comprised five significantly downregulated genes (*CARD17*, *LCN12*, *FUOM*, *LOC105374145*, and *LOC107984639*) and five significantly upregulated genes (*KCND3*, *LOC101928092*, *NLRP6*, *S100A8*, and *ELDR*) (Figure 4A). In addition, we identified five long intergenic non-coding (LINC) RNAs *LINC00539*, *LINC01103*, *LINC01765*, *LINC01792*, and *LINC02355*.

We identified 93 DEGs after treatment with GP-C (Table S2). The number of downregulated genes (62 genes) was twice that of the upregulated genes (31 genes) (Table S2). Similarly, among the top ten significant DEGs identified, the number of significantly downregulated genes (seven genes) was higher than that of the upregulated genes (three genes) (Figure 4B). The top downregulated genes were *LOC105370794*, *DDR2*, *TMEM200A*, *GIMD1*, *NMNAT3*, *NCKAP5*, and *LOC102723862*, whereas *TPH1*, *LOC107985155*, and *LTBP1*, were significantly upregulated. Moreover, four lncRNAs (*LINC00498*, *LINC00539*, *LINC01359*, and *LINC02355*) were found to be differentially expressed following GP-C treatment.

Of the 90 DEGs in the GP-CR treatment (Table S3), five genes, *LOC107984359*, *TMEM26*, *LOC105370794*, *LOC105376934*, and *NMUR2*, were identified as significantly downregulated, whereas three genes, *LOC107985495*, *LTBP1*, and *CAMKMT*, were identified as significantly upregulated (Figure 4C). Further, we identified four lncRNAs, *LINC00539*, *LINC00877*, *LINC02224*, and *LINC02355*, under GP-CR treatment (Table S3).

Under GP-all analysis, 176 DEGs were identified (Table S4). The most significantly upregulated genes were *SLC26A11*, *LTBP1*, *LOC105377548*, *TPH1*, and *CFAP70*, whereas three genes, *CRHR2*, *LOC107986720*, and *LOC105377128*, were significantly downregulated (Figure 4D). Three lncRNAs, *LINC00052*, *LINC02355*, and *LINC00539*, were identified as significant DEGs in GP-all (Table S4).

**Figure 5:**
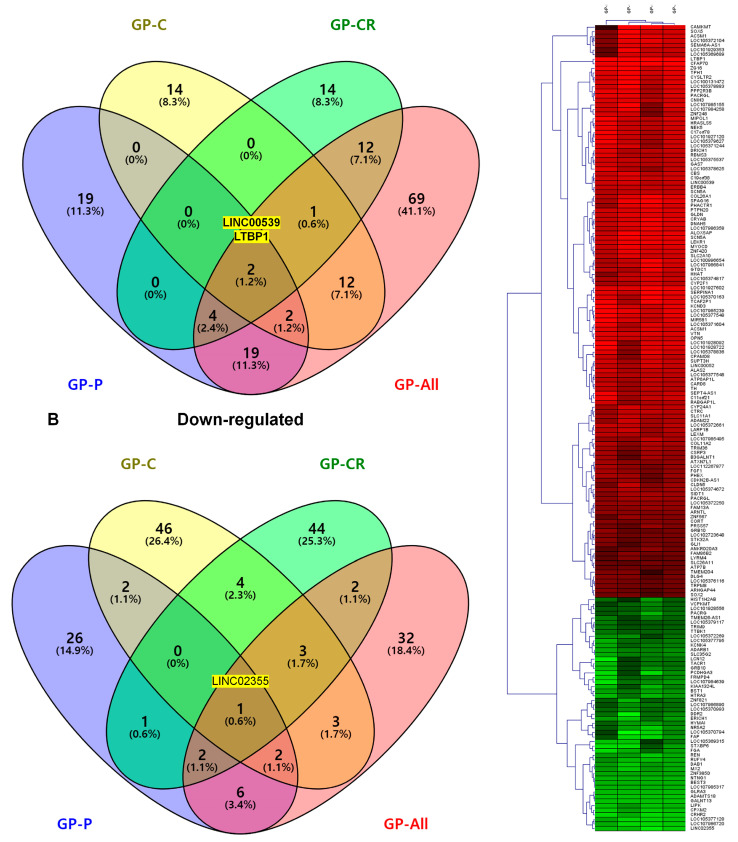
Comparison of the numbers of DEGs in four different conditions. Venn diagrams display the number of (**A**) upregulated genes and (**B**) downregulated genes among the four conditions. Yellow colored box indicates commonly identified transcripts in four conditions. (**C**) Heatmap shows the expression of the 176 DEGs identified in GP-all under the four different conditions. Red and green colors indicate up- and downregulated genes, respectively. DEG, differentially-expressed genes; GP, *Gynostemma pentaphyllum*.

We compared the number of DEGs identified under the four conditions. In total, 168 DEGs were highly upregulated (Figure 5A), while 174 were highly downregulated (Figure 5B). Of the 168 upregulated genes, *LTBP1* and *LINC00539* were commonly identified under all four conditions (Figure 5A). The expression of *LINC02355* was highly downregulated under all four conditions (Figure 4B). Many DEGs were condition-specific. For example, 69 upregulated and 32 downregulated genes were identified only under the GP-all condition. We examined the expression of the 176 DEGs identified from the GP-all condition in the other three conditions using a heatmap (Figure 5C). The heatmap showed that the expression of most DEGs identified in the GP-all condition was quite similar to that in the other three conditions.

### 3.4. Functional Classification of DEGs in Response to GP Extracts

**Figure 6:**
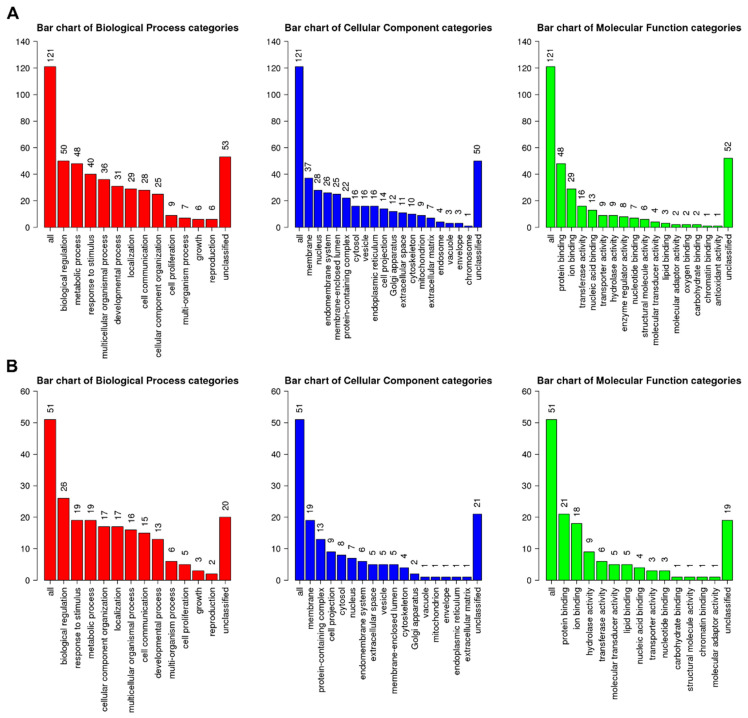
Summary of assigned gene ontology (GO) slim for 176 DEGs. The number of assigned (**A**) upregulated genes and (**B**) downregulated genes according to three categories for GO ontology, namely biological process, cellular component, and molecular function.

Based on the heatmap results, most DEGs identified in GP-all showed similar gene expression patterns. Therefore, we conducted a gene enrichment analysis using only the DEGs from GP-all. DEGs from GP-all were divided into upregulated and downregulated genes. Of the 125 upregulated genes, 121 were assigned GO term annotations. Genes assigned to biological regulation (50 genes) were most abundant, followed by those associated with metabolic process (48 genes) and response to stimulus (40 genes) when annotated by the biological process (Figure 6A). Genes associated with the membrane (37 genes) were most abundant, followed by those associated with the nucleus (28 genes) and endomembrane system (26 genes) when annotated by cellular components. Genes related to protein binding (48 genes) were most abundant, followed by ion binding (29 genes) and transferase activity (16 genes) when annotated by molecular function.

Of the 51 downregulated genes, a high number were assigned to biological processes (26 genes), responses to stimuli (19 genes), and metabolic processes (19 genes) (Figure 6B). Many downregulated genes were associated with membranes (19 genes), protein-containing complexes (13 genes), and cell projections (nine genes). Two GO terms, endosome (four genes) and chromosome (one gene), were identified only among the upregulated genes. Compared to the upregulated genes assigned to 16 GO terms according to molecular functions, the downregulated genes were assigned to only 13 GO terms. Three GO terms, antioxidant activity (one gene), oxygen binding (one gene), and enzyme regulator activity (eight genes), were identified only in the upregulated genes.

**Figure 7:**
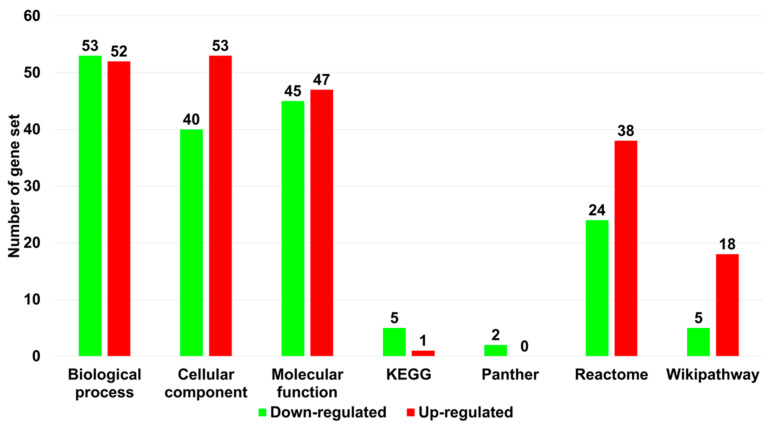
Enriched gene set for 176 DEGs based on several functional databases. To identify enriched gene sets, enrichment analyses were conducted based on gene ontology (biological process, cellular component, and molecular function), KEGG, Panther, Reactome, and Wikipathway using the WebGestalt program. Green and red colored bars indicate down- and upregulated genes, respectively. *p* < 0.05 was used as a cutoff.

To identify the enriched functions for the 176 DEGs identified from GP-all, we conducted gene set analyses using the WebGestalt program, which contains several databases. For this, 125 upregulated and 51 downregulated genes were subjected to gene set analysis (Tables S5 and S6). GO enrichment analyses showed a similar number of GO terms for up- (52 terms) and downregulated genes (53 terms) according to the biological processes (Figure 7). However, based on cellular components and molecular functions, the number of GO terms for upregulated genes was higher than that for downregulated genes.

**Figure 8:**
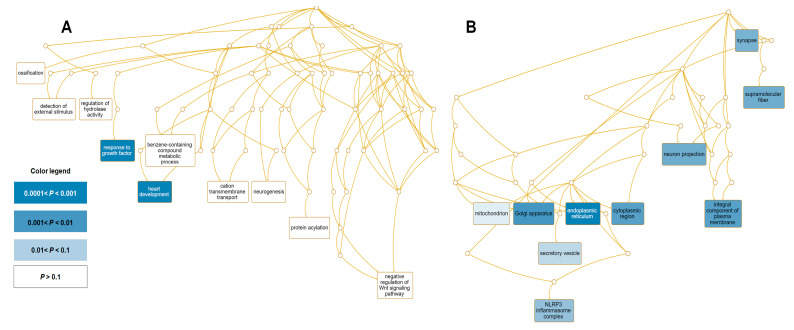
Significantly enriched GO terms for 125 upregulated genes. Directed acyclic graphs (DAGs) display the most significant GO terms for upregulated genes according to (**A**) biological process and (**B**) cellular component. The DAG displays the hierarchical relationship of identified GO terms. The blue colored boxes indicate the most significant GO terms. The significance is indicated by the color intensity.

For upregulated genes, two GO terms, response to growth factor (GO:0070848) and heart development (GO:0007507), were the most significant functional GO terms assigned to the biological processes (Figure 8A). The 11 genes assigned to growth factors were *LTBP1*, *MYOCD*, *VTN*, *SOX5*, *ERBB4*, *TH*, *CLDN5*, *FGF1*, *GRB10*, *TMEM204*, and *SOX2* (Table S7). The nine genes related to heart development were *LTBP1*, *SCN5A*, *DNAH5*, *MYOCD*, *ERBB4*, *TH*, *CLDN5*, *CSRP3*, and *GLI1*. Moreover, monooxygenase activity (GO:0004497)-associated GO term was the most significant molecular function, and four genes, *TPH1*, *CYP24A1*, *CYP2F1*, and *TH*, were involved (Table S7 and Figure 8B). Many upregulated genes were targeted to several cellular components, such as synapse (nine genes), supramolecular fiber (eight genes), neuron projection (10 genes), and integral components of the plasma membrane (12 genes), Golgi apparatus (11 genes), endoplasmic reticulum (15 genes), cytoplasmic region (six genes), and NLRP3 inflammasome complex (one gene) (Table S7).

**Figure 9:**
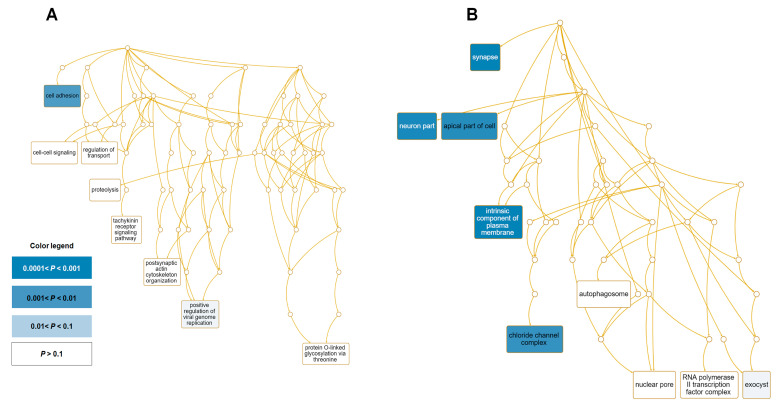
Significantly enriched GO terms for 51 downregulated genes. DAGs display the most significant GO terms for upregulated genes according to (**A**) biological process and (**B**) cellular component. The DAG displays the hierarchical relationship of identified GO terms. The blue colored box indicates the most significant GO terms. The significance is indicated by the color intensity.

Of the 51 downregulated genes, a GO enrichment analysis revealed that nine genes associated with cell adhesion (GO:0007155) were strongly downregulated (Table S8 and Figure 9A). In addition, two genes related to the positive regulation of viral genome replication (GO:0045070) were strongly downregulated. We found that many downregulated genes were localized to diverse cellular components, such as the synapse (seven genes), neuron part (eight genes), apical part of the cell (four genes), an intrinsic component of the plasma membrane (nine genes), chloride channel complex (two genes), and exocyst (one gene) (Figure 9B).

An enrichment analysis using the 125 upregulated genes in the KEGG database revealed that folate biosynthesis (hsa00790) was a significant metabolic pathway (Table S5). In contrast, among the 51 downregulated genes, five metabolic pathways, including neuroactive ligand–receptor interaction (hsa04080), renin–angiotensin system (hsa04614), maturity-onset diabetes of the young (hsa04950), nicotinate and nicotinamide metabolism (hsa00760), and mucin-type O-glycan biosynthesis (hsa00512) were significantly enriched (Table S6).

Enrichment analyses using the PANTHER database revealed that the circadian clock system (P00015) and vitamin D metabolism and activity pathways (P04396), were significantly enriched in the upregulated genes, while the plasminogen activating cascade (P00050) and axon guidance mediated by Slit/Robo (P00008) pathways were highly enriched in the downregulated genes.

Reactome pathway analyses identified 38 and 24 enriched pathways for up- and downregulated genes, respectively. For instance, molecules associated with elastic fibers (R-HSA-2129379), collagen chain trimerization (R-HSA-8948216), and elastic fiber formation (R-HSA-1566948) were identified in the upregulated genes, whereas post-translational protein modification (R-HSA-597592), post-translational modification: synthesis of GPI-anchored proteins (R-HSA-163125), and protein methylation (R-HSA-8876725) were identified in the downregulated genes.

Enrichment analysis against the Wikipathway database, providing biological pathways, revealed 18 and 5 enriched pathways for up- and downregulated genes, respectively. Among the upregulated genes were lung fibrosis (WP3624), dopaminergic neurogenesis (WP2855), biogenic amine synthesis (WP550), amino acid metabolism (WP3925), tryptophan metabolism (WP465), heme biosynthesis (WP561), and vitamin D metabolism (WP1531). Among the downregulated genes, splicing factor NOVA regulated synaptic proteins (WP4148), ACE Inhibitor pathway (WP554), and NAD+ biosynthetic pathways (WP3645) were identified.

## 4. Discussion

In this study, we established a mass production system for GP cells based on tissue culture and bioreactors and identified six metabolites in GP extracts. Three of the identified metabolites (uridine, adenosine, and guanosine) were nucleosides. A previous study suggested that guanosine and uridine have therapeutic roles in asthma and exert anti-inflammatory effects [34]. Furthermore, we identified three aromatic amino acids, tyrosine, phenylalanine, and tryptophan, in the extracts. Tryptophan and phenylalanine play important roles in the human diet. For example, tryptophan metabolism is associated with serotonin production, whereas phenylalanine is necessary for tyrosine production [35]. Notably, GP-C and GP-CR contained much higher amounts of the six metabolites than GP-P. This result suggested the enrichment of major metabolites in the extract from the cultured GP cells.

The effects of radiofrequency on plant growth have not yet been well-studied. A recent study showed that a weak radiofrequency magnetic field changed the cryptochrome-dependent plant growth response and gene expression in *Arabidopsis* [36]. We tested the possible applications of radiofrequency in plant cell culture. Similarly, we assumed that treatment with weak radiofrequency might alter the metabolites of GP cells; hence, treatment with the extract from such radiofrequency-treated GP cells may alter gene expression in HaCaT cells. However, we did not observe any significant differences in the gene expression profiles between GP-C and GP-CR, although the expression of some genes was condition-specific. We supposed that the radiofrequency treatment in our study might not have been high enough to change the metabolites of GP cells.

Based on the metabolites identified from GP extracts, it might be of interest to examine the possible effects of GP extracts on human skin cells. To this end, we compared the gene expression profiles of HaCaT cells after treatment with the three different GP extracts. The gene expression profiles in response to the treatments were similar. For example, genes highly upregulated in the GP-P condition were also upregulated in the other three conditions and vice versa. Therefore, the metabolites and their concentrations in the three GP extracts may be very similar, with minor differences. Based on these results, we concluded that the extracts from plant cell tissue culture and bioreactors could replace those derived from whole plants.

Most DEGs identified in GP-all showed similar expressions to the other three groups. Interestingly, we found that not only genes coding for proteins but also several lncRNAs were identified as DEGs after GP extract treatment. LncRNAs are non-coding RNA transcripts longer than 200 nucleotides in size, and at least 15,000 lncRNAs have been reported in genomic regions that do not include protein-coding genes in humans [37]. They also contain promoter- or enhancer-related RNA close to the gene region [37], and therefore, participate in a wide range of biological processes, including gene expression. In this study, we identified two upregulated (*LINC00052* and *LINC00539*) and one downregulated (*LINC02355*) lncRNAs. Several studies have demonstrated that *LINC00052* is involved in tumorigenesis, progression, and metastasis in several types of human cancers [38]. A previous study using gene expression data showed that *LINC00539* might be involved in the immune response against lung adenocarcinoma [39]. These results suggest that GP extract might be associated with the immune response to tumors by inducing the expression of *LINC00052* and *LINC00539*. However, the functional role of *LINC02355* has not been reported. Human tissue-specific transcriptome data showed that *LINC02355* was highly expressed in testis and thyroid tissues [40].

The most significantly upregulated gene under the four conditions was *LTBP1*. *LTBP1* is a member of the latent transforming growth factor β (TGFβ)-binding protein (LTBPs) family, which is composed of four proteins (LTBP1–LTBP4). LTBPs associate with microfibrils to anchor TGF-β in the extracellular matrix (ECM) and are required for the correct assembly of ECM components [41]. LTBP1 plays an important role in skin and bone ECM assembly and homeostasis [42].

GP extract induced the expression of 11 genes involved in the response to growth factors (GO:0070848). Of these, the *MYOCD* gene encoding myocardin plays an important role as a master regulator of smooth muscle gene expression [43]. *VTN* gene encodes vitronectin protein, a glycoprotein in the hemopexin family that is abundantly present in the serum of the ECM and bone [44]. In addition, two genes (*SOX2* and *SOX5*) are transcription factors in the SRY-box transcription factor (SOX) family, and their expression was highly upregulated by GP extract treatment. SOX family transcription factors are involved in diverse cellular developmental processes with functional redundancy [45]. *ERBB4* encodes the epidermal growth factor receptor-4 protein and is required for normal tissue development, such as the heart and nervous system, and tumor suppression [46]. *CLDN5* is a member of the claudin family and is an integral membrane protein specific to tight junctions that provide a protective paracellular barrier [47]. *FGF1*, which encodes fibroblast growth factor 1, plays a vital role in embryonic development, wound healing, neurogenesis, angiogenesis, and the control of type 2 diabetes mellitus [48]. *GRB10* is a member of the growth factor receptor-bound protein family and a negative regulator of insulin signaling and action [49]. *TMEM204* is a member of the transmembrane protein (TMEM) family and is required for angiogenesis and tumorigenesis [50].

We found that nine genes upregulated by the GP extract were involved in heart development (GO:0007507). Of these nine genes, five, namely *LTBP1*, *MYOCD*, *ERBB4*, *TH*, and *CLDN5*, were required for the response to growth factors (GO:0070848). Therefore, these five genes specifically function as growth factors in heart development. In addition to the other four genes associated with heart development, *SCN5A*, which encodes the alpha subunit of the main cardiac sodium channel, is associated with multiple cardiac disorders, such as cardiac conduction system dysfunction and dilated cardiomyopathy [51]. A previous study showed that mutations in the *DNAH5* gene encoding dynein cause primary ciliary dyskinesia with outer dynein arm defects [52]. Moreover, mutations in *CSRP3*, a member of the CSRP family containing the LIM domain, cause hypertrophic cardiomyopathy, a genetic cardiac disease, and the most common cause of sudden cardiac death [53]. Glioma-associated oncogene homolog 1 (*GLI1*) is a zinc finger transcription factor involved in the hedgehog (HH) signaling pathway, required for normal cell growth and differentiation [54]. In our study, a Wikipathway analysis revealed that the HH signaling pathway (WP47) was enriched in the genes upregulated by the GP extract. Our results are consistent with those of a previous study in which the aqueous extract of GP showed positive cardiovascular effects [55]. However, aberrant HH signaling due to truncated GLI1 can cause several human cancers [56].

GP extract has been widely used as an anticancer agent, and several studies have shown the inhibitory activity of GP extracts against cancer cells in vivo and in vitro [57]. In our study, several GP extract-upregulated genes, such as *ERBB4* and *GL1*, are associated with diverse cancers. The main compounds in GP extract were gypenosides, followed by sterols, flavonoids, and polysaccharides. Therefore, we hypothesized that GP gypenosides could play important roles as anticancer agents.

Reactome pathway analysis revealed that several genes associated with elastic fibers (R-HSA-2129379), collagen chain trimerization (R-HSA-8948216), and elastic fiber formation (R-HSA-1566948) were upregulated. Elastic fibers are important connective tissue components that provide elasticity and resilience to the skin, lungs, and blood vessels [58]. They consist of elastin and many elastin-associated microfibrillar proteins that assemble into a complex fiber network [58].

Aging is easily visible as changes in the skin. In normal human skin, the loss of elastic fibers with age results from the loss of skin thickness [59]. Moreover, the loss of skin elasticity with age is caused by the loss of fibroblast cells owing to a reduction in biosynthetic activity and modification of ECM macromolecules [59]. Many genes involved in the response to growth factors, such as *LTBP4* have also been associated with elastic fibers. The genes highly upregulated by the GP extract encode components of elastic fibers and the ECM. Treatment of human skin cells with GP extract may promote the expression of genes associated with growth factors. Moreover, an increase in growth factor proteins may prevent the aging of human skin.

Among the known metabolic pathways, two upregulated genes, *TH* and *TPH1* were associated with folate biosynthesis (hsa00790). *TH* encodes tyrosine hydroxylase, whereas *TPH1* encodes tryptophan hydroxylase 1. Moreover, *CYP24A1* encoding cytochrome P450 family 24 subfamily A member 1, is involved in vitamin D metabolism. Several studies have suggested that vitamin D and folate metabolism may be correlated with vascular health and human skin pigmentation [60]. The high expression of these three genes on treatment with the GP extract could cause the accumulation of vitamin D in the skin, and the active metabolites of vitamin D have a wide range of anti-aging and photoprotective effects on the skin [61].

In contrast, the most significant function of the downregulated genes was cell adhesion (GO:0007155). Cell adhesion molecules allow cells to adhere to each other and to the ECM [62]. Therefore, they play important roles in the formation and structural integrity of the epithelium of human skin [63]. Of the identified genes associated with cell adhesion, *DDR2* encoding discoidin domain receptor 2, a collagen receptor, regulates cell proliferation, and the absence of *DDR2* results in dwarfism in mouse mutants [64]. Thus, the downregulation of *DDR2* by the GP extract may have negative effects on cell adhesion. Another identified gene downregulated by GP extract was *ADAMTS18*, which is a member of the disintegrin metalloproteinase with thrombospondin domains (ADAMTS) family composed of 19 genes. ADAMTS genes participate in the assembly and degradation of ECM during development, morphogenesis, tissue repair, and remodeling [65]. Loss of *ADAMTS18* function promotes the growth, migration, and metastasis of melanomas [66]. Thus, the downregulation of *ADAMTS18* gene expression by GP extract may have a positive effect on cell adhesion.

Enriched cellular components in both up- and downregulated genes were associated with the synapse (GO:0045202), neuron projection (GO:0043005), neuron part (GO:0097458), and an intrinsic component of the plasma membrane (GO:0031226). As expected, the GP extract affected the expression of genes associated with the plasma membrane. Notably, many DEGs were associated with neurons. Consistent with our results, previous studies have shown that GP has positive effects on injured spinal motor neurons [67] and neuroprotective effects in a rat model of cardiopulmonary resuscitation [68].

Many upregulated genes were associated with several cell components, such as supramolecular fibers (GO:0099512), Golgi apparatus (GO:0005794), endoplasmic reticulum (GO:0005783), and cytoplasmic region (GO:0099568), and four downregulated genes, *DDR2*, *FAP*, *DAB1*, and *REN*, were specifically associated with the apical part of the cell (GO:0045177).

GP extract also induced the expression of genes involved in several metabolic pathways, such as amino acid metabolism, tryptophan metabolism, and heme biosynthesis, which are essential processes in human organs. For example, tryptophan metabolism is highly associated with inflammation, energy homeostasis, and brain function [69].

Taken together, our study provides comprehensive expression profiles of HaCaT cells in response to GP extracts and reveals many crucial biological processes that may be affected by GP extracts.

## 5. Conclusions

A wide range of effects of GP extracts has been reported in numerous studies. However, changes in the gene expression profile in human cells in response to GP extract have not yet been investigated. In this study, we identified six metabolites (uridine, adenosine, guanosine, tyrosine, phenylalanine, and tryptophan) in GP extracts, and compared the transcriptome profiles of HaCaT cells in response to treatment with GP extracts obtained through three independent methods from plants, calli, and calli after radiofrequency treatment. Some genes showed condition-specific expression; however, most DEGs identified in the GP-all condition exhibited a similar gene expression in the three GP extracts. The most significantly upregulated gene under the four different conditions was *LTBP1*, which is required for skin and bone ECM assembly as well as homeostasis. Moreover, we identified two upregulated (*LINC00052* and *LINC00539*) and one downregulated (*LINC02355*) lncRNAs. On combining the data, we identified 125 upregulated and 51 downregulated genes in response to all extracts. The enriched functions of the 125 upregulated genes were associated with response to growth factors and heart development. Moreover, genes encoding components of elastic fibers and ECM were upregulated by the GP extract. Some upregulated genes, such as *ERBB4* and *GL1*, are also associated with diverse cancers. Furthermore, the upregulation of genes related to folate biosynthesis and vitamin D metabolism suggests the functional roles of the extracts in anti-aging and photoprotective effects on the skin. In contrast, the most enriched function among the downregulated genes was cell adhesion. Moreover, many DEGs were targeted to synapse and neuron projections, indicating their possible roles in neuroprotection. In summary, we provide a comprehensive overview of gene expression changes in HaCaT cells after treatment with GP extracts and reveal their functional roles in anti-aging and photoprotective effects on the skin using RNA sequencing.

## Figures and Tables

**Table 1:** List of the 12 HaCaT samples used for RNA sequencing.

Index	Condition	Treatment	Library Name	Replicate
1	Control	Distilled water	DW-1	1
2	Control	Distilled water	DW-2	2
3	Control	Distilled water	DW-3	3
4	GP-P	GP extract from plants	GP-P1	1
5	GP-P	GP extract from plants	GP-P2	2
6	GP-P	GP extract from plants	GP-P3	3
7	GP-C	GP extract from callus	GP_C1	1
8	GP-C	GP extract from callus	GP_C2	2
9	GP-C	GP extract from callus	GP_C3	3
10	GP-CR	GP extract from callus treated with radiofrequency	GP_CR1	1
11	GP-CR	GP extract from callus treated with radiofrequency	GP_CR2	2
12	GP-CR	GP extract from callus treated with radiofrequency	GP_CR3	3

HaCaT cells were treated with distilled water (control) or extracts of GP. Three biological replicates were used for each condition. GP, *Gynostemma pentaphyllum.*

**Table 2:** Summary of the raw data obtained by RNA sequencing.

Library Name	Total Reads	Total Read (bp)	GC (%)	AT (%)	Q20 (%)	Q30 (%)
DW-1	42,923,432	4,335,266,632	50.81	49.19	98.04	94.69
DW-2	35,861,160	3,621,977,160	50.51	49.49	98.33	95.15
DW-3	36,625,918	3,699,217,718	51.06	48.94	98.22	95.07
GP-P1	40,523,746	4,092,898,346	51.19	48.81	98.08	94.72
GP-P2	38,146,816	3,852,828,416	50.72	49.28	98.28	95.11
GP-P3	49,818,356	5,031,653,956	50.98	49.02	98.29	95.16
GP_C1	35,192,798	3,554,472,598	51	49	98.24	95.06
GP_C2	33,870,946	3,420,965,546	50.7	49.3	98.15	94.85
GP_C3	44,721,472	4,516,868,672	50.82	49.18	98.23	94.98
GP_CR1	34,605,546	3,495,160,146	50.48	49.52	98.05	94.63
GP_CR2	42,126,968	4,254,823,768	50.9	49.1	98.08	94.89
GP_CR3	32,703,588	3,303,062,388	50.73	49.27	98.22	95.02

bp, base pair; Q20 and Q30, quality scores of 20 and 30, respectively.

## Data Availability

The raw data were deposited in the NCBI SRA database with the following accession numbers: SRR22342813–SRR22342824.

## Supplementary Materials

The following supporting information can be downloaded at https://www.mdpi.com/article/10.3390/life13020423/s1: Table S1: List of differentially expressed genes by the GP extract from the plant; Table S2: List of differentially expressed genes by the GP extract from the callus; Table S3: List of differentially expressed genes by the GP extract from the callus treated with radiofrequency; Table S4: List of differentially expressed genes by the three GP extracts; Table S5: Enriched functions in 125 upregulated genes; Table S6: Enriched functions in 51 downregulated genes; Table S7: Representative functions enriched in 125 upregulated genes and related gene list; Table S8: Representative functions enriched in 51 downregulated genes and related gene list. Figure S1: HPLC chromatograms of *Gynostemma pentaphyllum* and tandem mass spectrometry of each peak fraction.
