# Supplementary material for: Comparative Transcriptome Profiles of Human HaCaT Cells in Response to Gynostemma pentaphyllum Extracts Obtained Using Three Independent Methods by RNA Sequencing
Source: Life (Basel). 2023 Feb 2;13(2):423. doi: 10.3390/life13020423 (PMC9961609; doi:10.3390/life13020423)
Supplement: Supplementary file 1 [file life-13-00423-s001.zip › Figure S1.pdf]

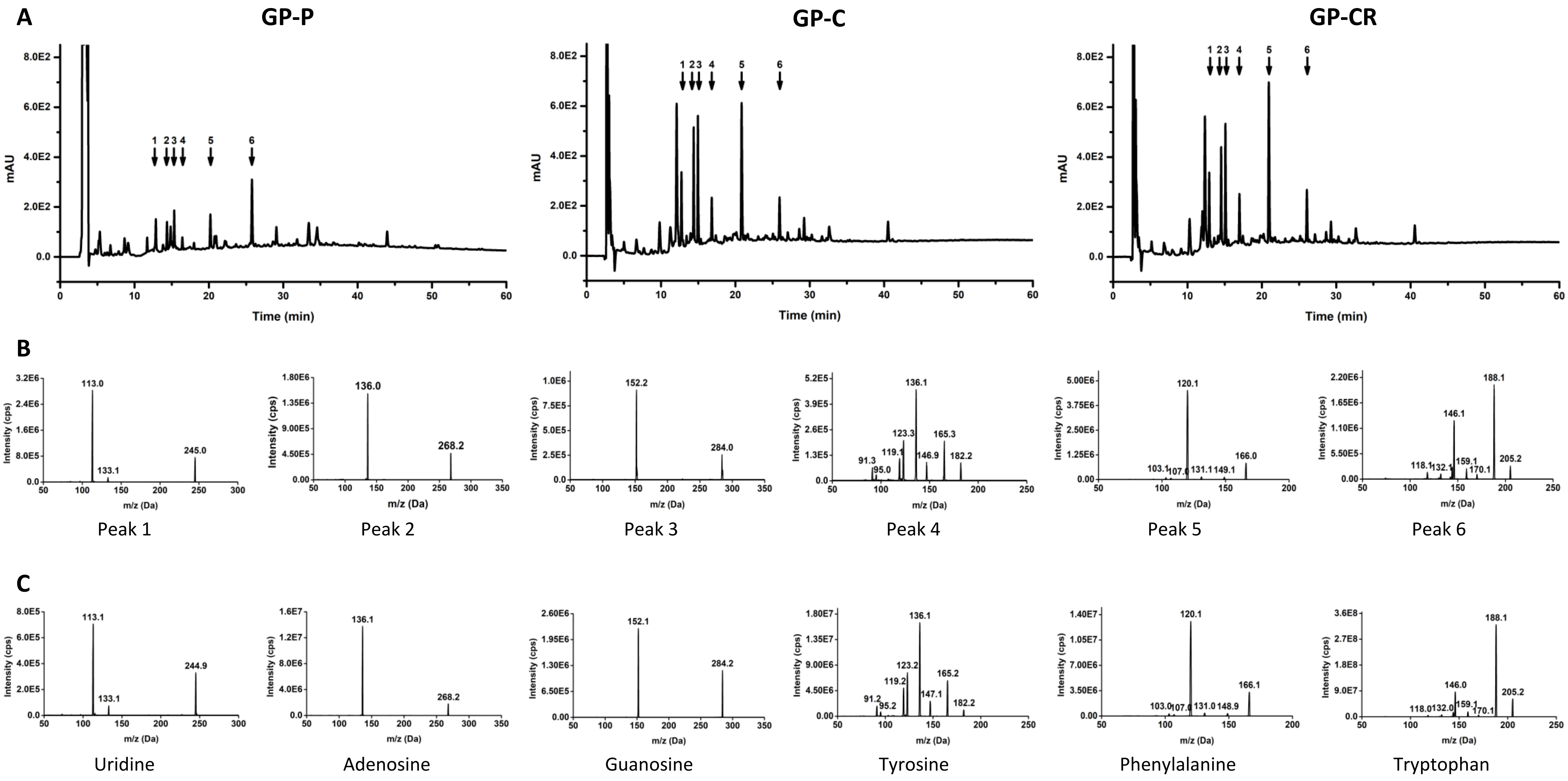

Figure S1. HPLC chromatograms of *Gynostemma pentaphyllum* and tandem mass spectrometry of each peak fraction. (A) HPLC chromatograms of GP-P, GP-C, and GP-CR. (B) Tandem mass spectrometry of each peak fraction (Peak 1~ 6). (C) Tandem mass spectrometry of each authentic compound (Peak 1; Uridine, Peak 2; Adenosine, Peak 3; Guanosine, Peak 4; Tyrosine, Peak 5; Phenylalanine, Peak 6; Tryptophan).
